# Complete genome sequences of cluster F1 and cluster B1 *Mycobacterium smegmatis* phages Karhdo and Basato

**DOI:** 10.1128/mra.00938-23

**Published:** 2023-12-05

**Authors:** Jireh Kim, Carlos Herrera, Wai Yan Aung, Gino Pablo Gonzales Boyles, Carmina Chavez, Mona Cibulka, Emma Foley, Jose Guerra, Disha Byadarahalli Mohan Kumar, Willow Levrant, Lewis Lim, Jose Llanes, Zachary Kamuela O'Brien, Art Pagaduan, Justin Aaron Richardson, Khristian Rosales, James Schrecengost, Tommy Shin, Gabriel Strong-Lundquist, Winnie Tat, Fritz Vanderford, Isabelle Vrinceanu, Vicky Wang, Stephanie Yang, Christy Strong, Philippos K. Tsourkas, Kurt Regner

**Affiliations:** 1 School of Life Sciences, University of Nevada, Las Vegas, Nevada, USA; 2 School of Medicine and Public Health, University of Wisconsin, Madison, Wisconsin, USA; Department of Biology, Queens College, Queens, New York, USA

**Keywords:** mycobacteria, bacteriophages, actinobacteriophage

## Abstract

We present the complete genome sequences of *Mycobacterium smegmatis* phages Karhdo and Basato, isolated in Clark County, Nevada. The phages were isolated and annotated by students enrolled in undergraduate research courses over two semesters at the University of Nevada, Las Vegas.

## ANNOUNCEMENT

Karhdo (36.131376 N, 115.240078 W) was isolated from compost, and Basato (35.985570 N, 115.123587 W) from a basil and tomato planter, both at private residences. Soil samples were incubated with enrichment broth, shaken (250 rpm, 2 h) at room temperature, followed by centrifugation and filter sterilization (0.22 µm) of the supernatant. Using *Mycobacterium smegmatis* mc^2^ 155 as the host, phages were considered pure after three rounds of plaque assays produced consistent plaque morphologies (1). Genomic DNA was isolated (Phage DNA Isolation Kit, Norgen Biotek), and samples were sequenced using the Illumina MiSeq System (v3 reagents) to yield 150 bp single-end reads with the reported coverage (Table 1). The total reads for Kardho and Basato were 1,205,997 and 210,277, respectively. The reads were quality trimmed and assembled *de novo* using Newbler (v. 2.9, 454 Life Science) to generate a single contig. Consed (v. 29) assessed accuracy, sequence completion, and phage genomic termini (2). For Transmission Electron Microscopy (TEM), 10 µLl of high titer (~10^10^) lysate was added to copper 300 mesh grids (Ted Pella, Inc.) and stained with 1% phosphotungstate (Electron Microscopy Services). TEM was performed at 120 keV with a JEOL JEM-1400 Plus. Images were obtained with DigitalMicrograph with a Gata Orius SC1000 CCD camera.

**TABLE 1:** Phage GenBank and SRA accession numbers and genome assembly results

Phage name	GenBank accession no.	SRA accession no.	Average coverage (✕)	Cluster and subcluster	Genome length (bp)	GC content (%)	No. of genes
Basato	OR159661.1	SRX21748117	439✕	B B1	68,518	66.5%	100
Karhdo	OR159669.1	SRX21748113	3421✕	F F1	55,379	61.5%	100

The putative genes of Karhdo and Basato were identified from FASTA files using DNA Master (v5.38.8) and Phage Commander, which retrieves query results from Glimmer (v3.02b), GeneMark (v2.5), GeneMark.hmm (v3.25), GeneMarkS (v4.28), GeneMark with Heuristics (v3.25), GeneMarkS2, RAST (v2.0), MetaGene, and Aragorn for tRNAs (3 – 13). The annotation program Prokka (v1.14.6), which uses Prodigal (v2.6.3), was also used (14, 15). The putative genes and start codons were evaluated as described (16). The putative protein functions were assigned using Protein BLAST, CD-Search, and HHpred, with the E-value cutoffs of 10^−7^, 0.001, and 0.001, respectively (17 – 19). Deep TMHMM (v1.0.24) and SOSUI were used to identify transmembrane domains (20, 21). Phage clusters and subclusters were determined with Phamerator (22), following protocols in reference (23). The default settings were used for all the software listed unless specified otherwise.

**Fig 1:**
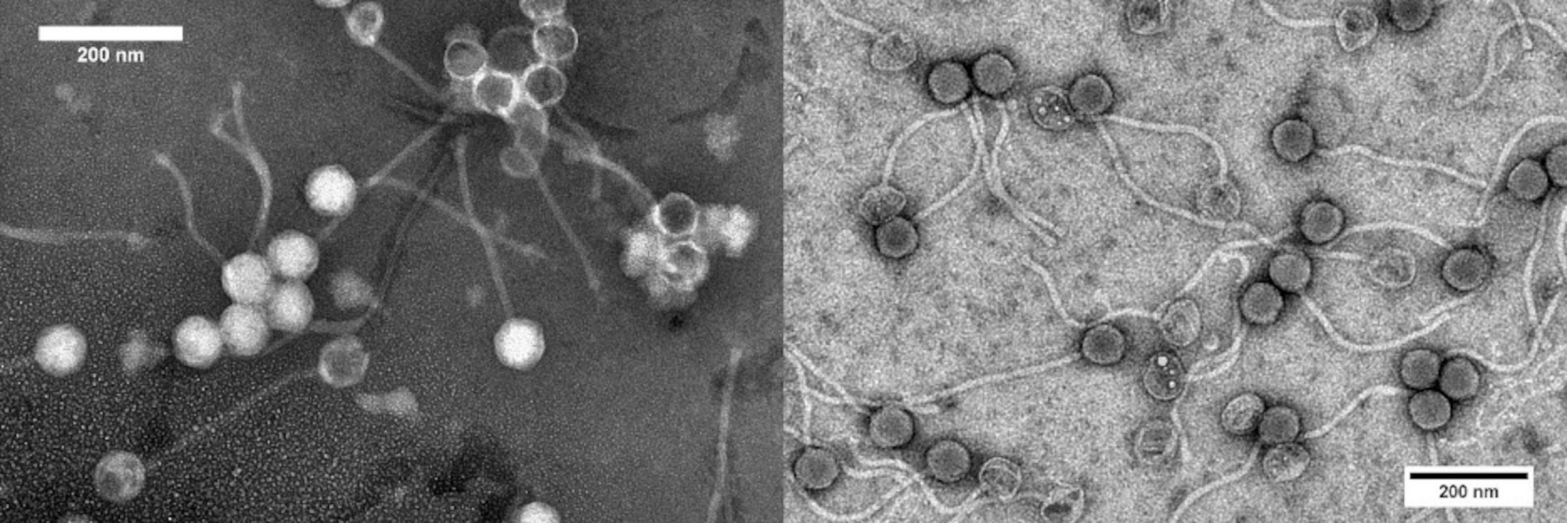
Transmission electron microscopy (TEM) of *Mycobacterium smegmatis* mc^2^ 155 bacteriophages Karhdo (left panel) and Basato (right panel). Samples were negatively stained with 10 mL of 1% phosphotungstate. Imaging was performed at 120 keV on JEOL-JEM-1400 Plus, Electron Microscopy Core Laboratory, University of Utah. Images captured by DigitalMicrograph with Gatan Orius SC1000 CCD camera.

Kardho plaques were 0.5 cm in diameter, while Basato plaques varied in diameter from 0.04 to 0.4 cm. Karhdo and Basato both display siphovirus morphology with recorded capsid diameters and tail lengths of 83 nm and 316 nm for Karhdo, and 73 nm and 306 nm for Basato, respectively (Fig. 1). Accession numbers and genome assembly results are listed in Table 1. Karhdo (cloudy temperate plaques), belongs to subcluster F1, while the Basato (clear lytic plaques) belongs to subcluster B1. Both phages have 100 genes. Karhdo has 41 genes with assigned protein functions, including a glycosyltransferase (genes 97 and 99), which has only been identified in cluster F phages. Basato has 29 genes with assigned protein functions. A programmed translational +1 frameshift in the tail assembly chaperone was found in Karhdo, and was annotated appropriately (3). Basato lacked a programmed translational frameshift. A complete list of genes and functions for both phages is available at The Actinobacteriophage Database (https://phagesdb.org).

## Data Availability

GenBank and SRA accession numbers are listed in Table 1.
